# Tryptophan confers resistance to SDS-associated cell membrane stress in *Saccharomyces cerevisiae*

**DOI:** 10.1371/journal.pone.0199484

**Published:** 2019-03-11

**Authors:** Lea Schroeder, Amy E. Ikui

**Affiliations:** Department of Biology, Brooklyn College, City University of New York, Brooklyn, New York, The United States of America; CNR, ITALY

## Abstract

Sodium dodecyl sulfate is a detergent that disrupts cell membranes, activates cell wall integrity signaling and restricts cell growth in *Saccharomyces cerevisiae*. However, the underlying mechanism of how sodium dodecyl sulfate inhibits cell growth is not fully understood. Previously, we have shown that deletion of the *MCK1* gene leads to sensitivity to sodium dodecyl sulfate; thus, we implemented a suppressor gene screening revealing that the overexpression of *TAT2* tryptophan permease rescues cell growth in sodium dodecyl sulfate-treated *Δmck1* cells. Therefore, we questioned the involvement of tryptophan in the response to sodium dodecyl sulfate treatment. In this work, we show that *trp1-1* cells have a disadvantage in the response to sodium dodecyl sulfate compared to auxotrophy for adenine, histidine, leucine or uracil when cells are grown on rich media. While also critical in the response to tea tree oil, *TRP1* does not avert growth inhibition due to other cell wall/membrane perturbations that activate cell wall integrity signaling such as Calcofluor White, Congo Red or heat stress. This implicates a distinction from the cell wall integrity pathway and suggests specificity to membrane stress as opposed to cell wall stress. We discovered that tyrosine biosynthesis is also essential upon sodium dodecyl sulfate perturbation whereas phenylalanine biosynthesis appears dispensable. Finally, we observe enhanced tryptophan import within minutes upon exposure to sodium dodecyl sulfate indicating that these cells are not starved for tryptophan. In summary, we conclude that internal concentration of tryptophan and tyrosine makes cells more resistant to detergent such as sodium dodecyl sulfate.

## Introduction

In the wild, yeast cells experience a variety of external conditions that cause stress, such as changes in resource availability, temperature, osmotic fluctuations, oxidation, noxious chemicals, pressure and physical stress. The yeast cell wall and plasma membrane are the first defensive structures against external stress and are essential to acclimate to these conditions. In general, any perturbation that disrupts the cell wall or membrane function activates a multifactorial stress response in *Saccharomyces cerevisiae* [1, 2].

Sodium Dodecyl Sulfate (SDS) is a common household detergent that permeates cell membranes [3,4], activates a stress response including Cell Wall Integrity (CWI) signaling and restricts cell growth [5,6]. The CWI pathway is a kinase cascade that responds to cell wall/membrane perturbations in order to maintain cell integrity in yeast [1,2]. Chemicals that damage the yeast cell wall or membrane such as SDS [5,6], Calcofluor White (CFW) [7], Congo Red (CR) [8] and Tea Tree Oil (TTO) [9] or by growth at elevated temperatures [10] trigger the CWI pathway.

*MCK1*, the yeast homologue of the mammalian Glycogen Synthase Kinase-3 (GSK-3) [11,12] is involved in a variety of stress response activities. Mck1p maintains genome integrity in response to DNA damage [13,14] and is involved in the transcriptional regulation of stress response genes [15,16]. In addition, Mck1p is a downstream effector of CWI signaling activated by high temperature, osmotic stress or calcium stress [17,18]. Deletion of *MCK1* causes hypersensitivity to SDS [16,18]. We previously found that SDS induces cell cycle arrest during G1 phase via Mck1p [16]. In order to understand the mechanism of cell growth inhibition by SDS, we implemented a suppressor gene screening using *Δmck1* cells in the presence of SDS. The screen revealed that overexpression of *TAT2* tryptophan permease rescued cell growth in SDS-treated *Δmck1* cells.

The high affinity tryptophan permease, Tat2p (Tryptophan Amino acid Transporter), is a constitutive permease regulated by the concentration of tryptophan in the media [19]. The appropriate function and localization of Tat2p and/or the ability to biosynthesize tryptophan is required for yeast to survive under a variety of stresses. In particular, perturbations that affect membrane stability may have strong auxotrophic requirements for tryptophan. For example, yeast cells experiencing high pressure [20], a deficiency in ergosterol (yeast version of cholesterol) [21–23], organic acid stress [24] or ethanol stress endure alterations to their membranes [25–28]. Furthermore, *TAT2* overexpression is a requirement for cell growth under high pressure [20] and is required for proper ergosterol localization [23]. It was also found that tryptophan supplement aids in the response to organic acid stress [24]. These previous findings raise the possibility that tryptophan itself exhibits protection from membrane disruptions. In addition to these cell wall/membrane related stresses, it has been suggested that internal tryptophan levels influence growth recovery post DNA damage [29,30].

Our suppressor gene screening revealed that *TAT2* is linked to tolerance towards membrane stress; we therefore questioned the involvement of tryptophan in the recovery of cell growth using SDS, which directly perturbs cell membranes [3,4]. In this work, we show that SDS-induced growth inhibition can be overcome with exogenous tryptophan or tryptophan prototrophy. We found that tryptophan prototrophy exhibits protection from growth inhibition due to particular cell wall/membrane damaging agents that activate the CWI pathway, but not all treatments, suggesting that the need for tryptophan is autonomous from CWI activity. In addition to tryptophan biosynthesis, we show that tyrosine biosynthesis is also necessary for tolerance to SDS stress. Additionally, we determine that tryptophan import is not disrupted by SDS exposure but enhanced. These results provide a strong connection to tryptophan and tyrosine in the protection from plasma membrane damage that is not due to general nutrient starvation and is independent of CWI signaling.

## Results and discussion

### Tryptophan availability recovers sensitivity to SDS

**Fig 1:**
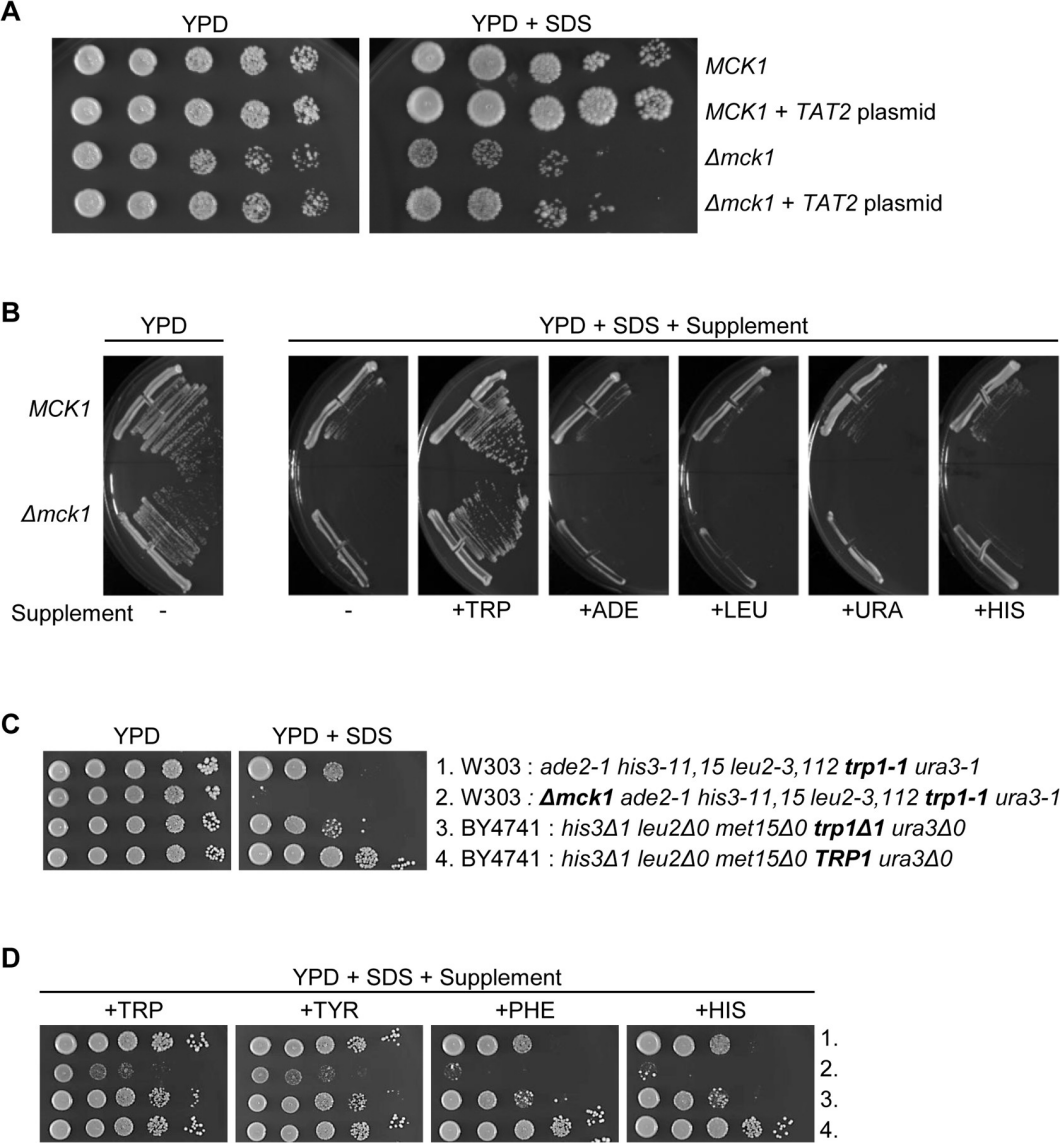
*TAT2*/Tryptophan rescues SDS sensitivity in *Δmck1* cells. (A) W303 *MCK1* cells (*his3-11*,*15 leu2-3*,*112 trp1-1 ura3-1*) and isogenic *Δmck1* cells transformed with *TAT2*/pRS425 plasmid were 5-fold serially diluted onto YPD or YPD plus 0.0075% SDS. (B) The same strains, *MCK1* or *Δmck1*, used in A were struck on YPD or YPD containing 0.0075% SDS and supplemented on top with excess tryptophan (+TRP), adenine (+ADE), leucine (+LEU), uracil (+URA) or histidine (+HIS). (C) The indicated strains were 10-fold serially diluted onto YPD or YPD plus 0.0075% SDS and (D) containing additional tryptophan (+TRP), tyrosine (+TYR), phenylalanine (+PHE) or histidine (+HIS).

To affirm the rescue of *Δmck1* sensitivity to SDS with *TAT2*, we cloned *TAT2* into a pRS425 high copy plasmid and asked if *TAT2* alone rescues SDS-induced cell growth inhibition in *Δmck1* cells. Indeed, we found that the *TAT2* expressing plasmid conferred rescue in both *Δmck1* and *MCK1* W303 cells on rich media plates (YPD) in the presence of SDS (Fig 1A). We also found that the *TAT2* plasmid did not rescue *Δmck1* sensitivity to SDS on synthetic media plates (SD) (S1 Fig) indicating that *TAT2* plays a role in the response to membrane stress when excess amount of amino acids and/or other components in rich media are available. It is known that during times of stress or nutrient starvation, Tat2p is sorted to the vacuole for degradation and then tryptophan uptake is maintained by Gap1p, the General Amino acid Permease [31–34]. It is possible that *TAT2* is not retained on the cell membrane when cells are grown on synthetic media therefore causing growth defects in the presence of SDS in spite of the excess amount of *TAT2* expression. This may explain why the SDS-induced growth inhibition in *Δmck1* cells was only mildly rescued by *TAT2* overexpression.

To support the idea that cell growth sensitivity to SDS recovered by *TAT2* overexpression is due to tryptophan availability, we also observed that exogenous tryptophan recovered growth in both *Δmck1* and *MCK1* cells in the W303 background when we supplemented YPD plates containing SDS with excess tryptophan (Fig 1B). However, cell growth was still inhibited by SDS with the addition of exogenous adenine, leucine, uracil or histidine suggesting that recovery of SDS-induced growth inhibition is specific to tryptophan (Fig 1B).

W303 and BY4741 are two commonly used laboratory strains of *S*. *cerevisiae*. It has been shown that BY4741 cells that are auxotrophic for tryptophan are sensitive to SDS-induced cell membrane stress in liquid culture [35]. We expand these results by comparing BY4741 cells containing or lacking functional *TRP1* in a serial dilution assay. We found that the presence of wild type *TRP1* in BY4741 cells confers resistance to SDS-induced growth inhibition on YPD plates containing SDS (Fig 1C, rows 3 and 4). *Δmck1* strain in W303 background was used as a control in these experiments and as expected, growth in the *Δmck1* cells was severely inhibited in the presence of SDS compared to wild type *MCK1* cells (Fig 1C, rows 1 and 2).

In addition, we tested if excess tryptophan also rescues cell growth sensitivity to SDS in BY4741 cells by comparing the same cell types used in Fig 1C on YPD plus SDS plates containing additional tryptophan, tyrosine, phenylalanine or histidine. Tyrosine and phenylalanine were used as a comparison because they are aromatic amino acids made by the same pathway that synthesizes tryptophan. The aromatic amino acid, histidine, was also used as a control because it is made by a separate pathway from tryptophan. Fig 1D shows that growth in the BY4741 cells disrupted for *TRP1* was improved in the presence of SDS when excess tryptophan was available (Fig 1D, rows 3 and 4). We show that excess tyrosine also rescued SDS sensitivity in BY4741 cells whereas phenylalanine and histidine did not. The W303 tryptophan auxotroph cells, *Δmck1* and *MCK1*, showed the same trend as the BY4741 cells (Fig 1D, rows 1 and 2). This confirms our previous conclusion that *MCK1* contributes to the SDS related stress response in a way that might be autonomous from tryptophan availability. These data show that, independent of strain background, cells harboring a nonfunctional *TRP1* are able to grow in the presence of SDS if sufficient amounts of tryptophan or tyrosine are externally available, further indicating the significance of *TRP1* prototrophy for SDS resistance in multiple yeast backgrounds.

### Cell growth inhibition by SDS is dependent on tryptophan synthesis

**Fig 2:**
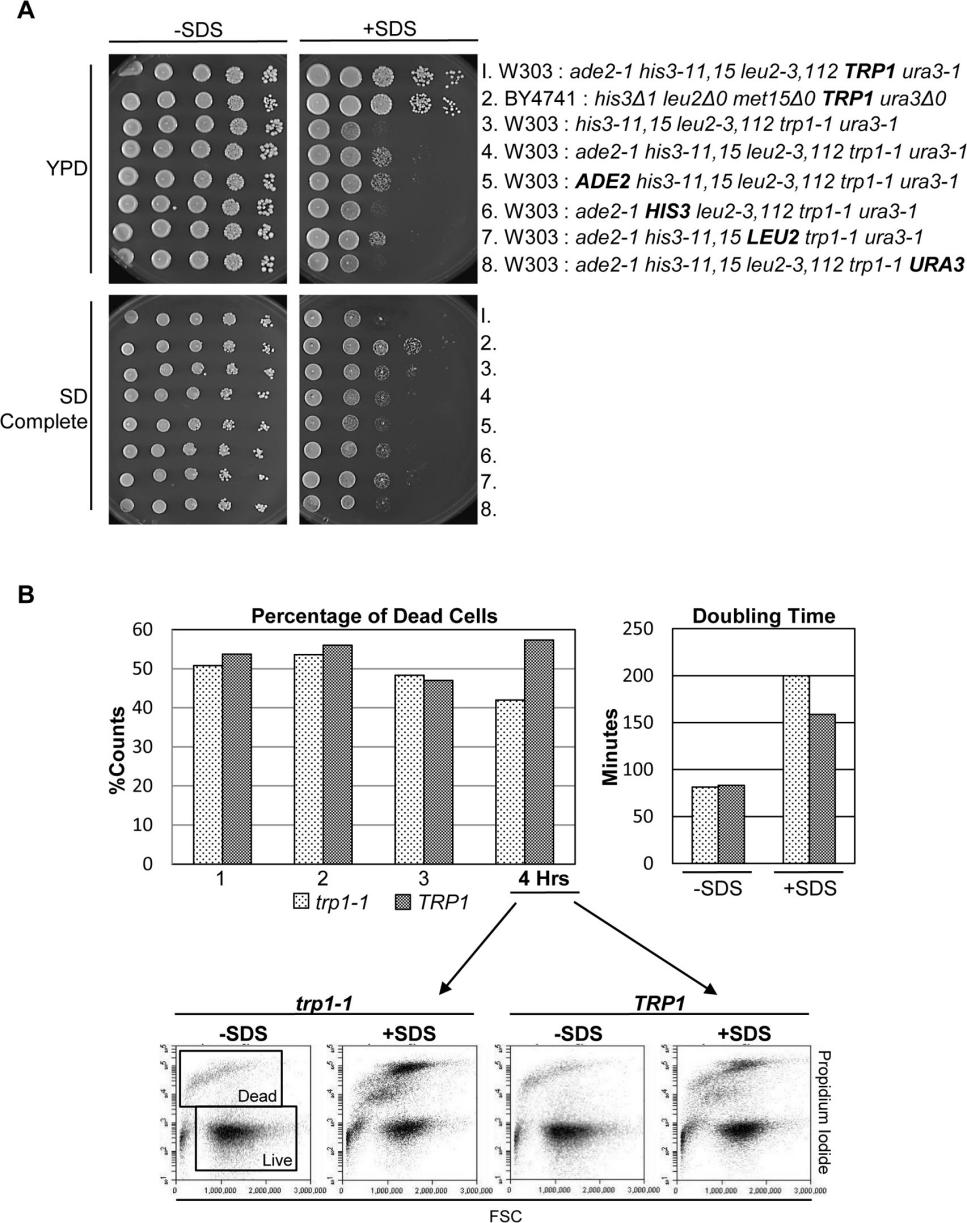
*TRP1* prototrophy confers growth advantage in the presence of SDS. (A) The indicated cells were 10-fold serially diluted onto YPD or SD plates with or without 0.0075% SDS. (B) Log phase cells of the genotype *ade2-1 his3-11*,*15 leu2-3*,*112 trp1-1 ura3-1* (*trp1-1*) and isogenic *TRP1* cells (*TRP1*) were grown in YPD with or without SDS (0.001%). Every hour for 4 hours the optical density (OD) was read and an aliquot of cells was stained with propidium iodide and analyzed by FACS. The bottom row is an example of the FACS readings at the 4 hour time-point and the gating used to determine live vs dead cells. The graph on the left indicates the percentage of dead cells. The graph on the right indicates the doubling times as the slope of the line determined by plotting log2 of the optical density reading vs. time.

These results prompted us to test further the prototrophic requirements for SDS resistance. W303 cells that are auxotrophic for all of the markers, *ade2-1*, *his3-11*,*15*, *leu2-3*,*112*, *trp1-1* and *ura3-1*, showed growth inhibition on YPD plates in the presence of SDS (Fig 2A, top, row 4). The matched cells harboring a wild type copy of *TRP1* showed robust growth on YPD plus SDS (Fig 2A, top, row 1). W303 cells that were prototrophic for any other single nutrient indicated besides tryptophan did not recover cell viability on rich media plates containing SDS (Fig 2A, top, rows 5–8). However, the presence or absence of *TRP1* did not affect growth on SD plates containing SDS (Fig 2A, bottom, rows 1 and 4) suggesting that components in YPD media enhance viability in *TRP1* prototrophic cells in the presence of SDS. BY4741 cells that are prototrophic for *TRP1* grew robustly at this concentration of SDS on both rich and synthetic plates (Fig 2A, row 2). We conclude that on YPD plates, *trp1* auxotrophy has a more adverse effect to cells compromised with SDS than auxotrophy for adenine, histidine, leucine or uracil and is an additional indication that other nutrients in peptone or yeast extract together with tryptophan may play a role in growth recovery when cells are treated with SDS.

To confirm these results, we compared the growth and viability of cells containing the 5 auxotrophic markers (*ade2-1*, *his3-11*,*15*, *leu2-3*,*112*, *trp1-1* and *ura3-1)*, named *trp1-1*, with isogenic counterpart cells containing wild type *TRP1*, named *TRP1*, in liquid culture (Fig 2B). Cells were collected every hour for 4 hours in the presence or absence of SDS. Cells were analyzed by FACS with propidium iodide staining in order to distinguish live versus dead cells. After 4 hours of incubation in SDS, there was a fewer proportion of dead cells in the sample containing wild type *TRP1* (dark gray) vs *trp1-1* (light gray) (Fig 2B, left). The cell doubling time was considerably faster in the *TRP1* cells than in the *trp1-1* cells (Fig 2B, right). These outcomes promote the idea, that wild type *TRP1* is beneficial for resistance to SDS treatment in YPD media.

**Fig 3:**
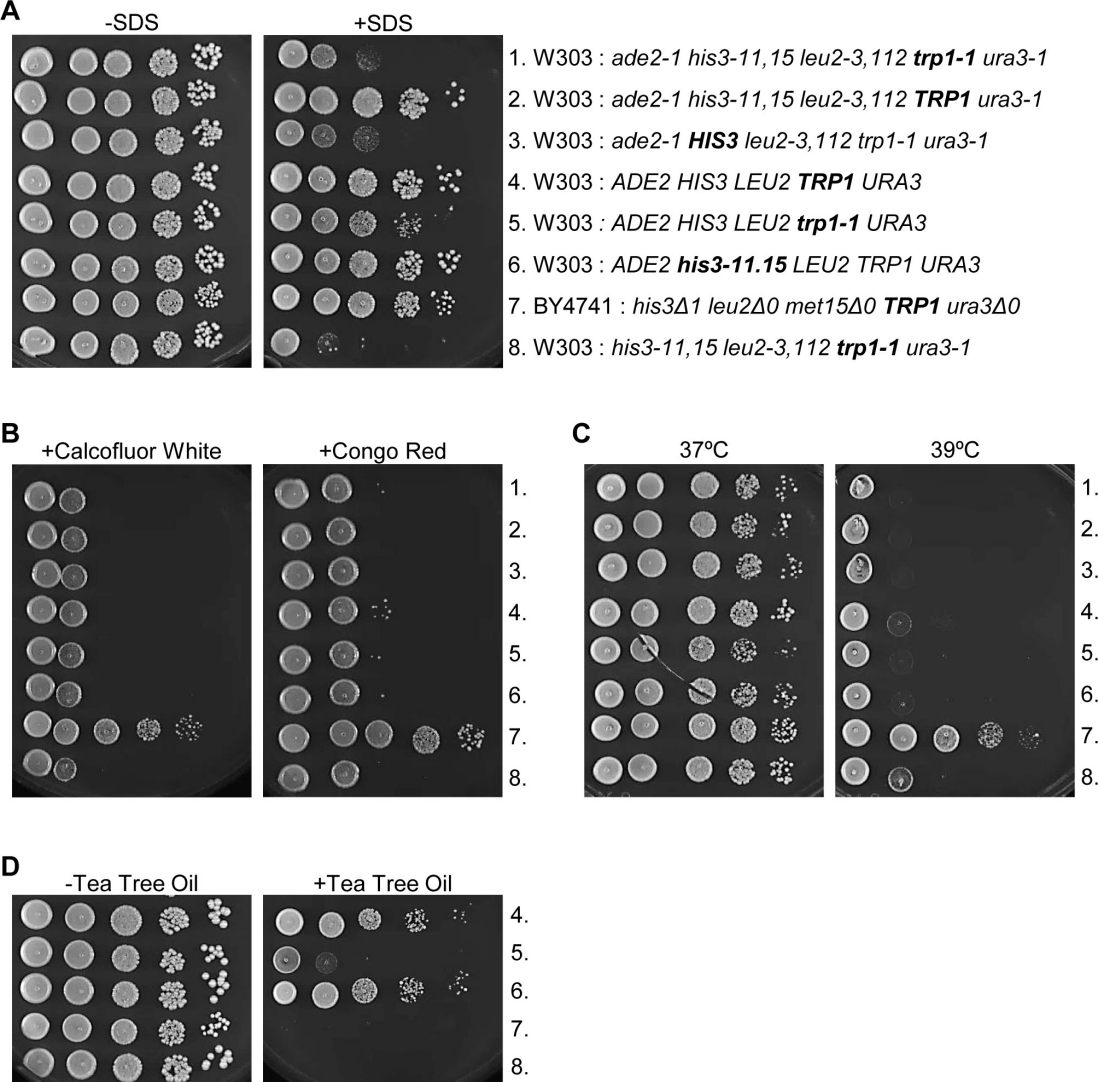
*TRP1* prototrophy recovers growth sensitivity due to some cell wall/membrane damaging treatments but not all. The indicated yeast cells were 10-fold serially diluted onto YPD (A) with or without 0.0075% SDS, (B) containing 10ug/ml Calcofluor White or 10ug/ml Congo Red, (C) incubated at 37°C or 39°C or (D) containing 0.15% Tea Tree Oil.

We also studied cells that are prototrophic for all the markers, *ADE2*, *HIS3*, *LEU2*, *TRP1* and *URA3* (Fig 3A, row 4). Fig 3A shows that, in general, prototrophic cells grow better in the presence of SDS than cells containing multiple auxotrophies (Fig 3A, rows 1 and 4). However, prototrophic cells harboring only a *trp1-1* mutation were sicker in the presence of SDS than cells harboring only a *his3-11*,*15* mutation (Fig 3A, rows 5 and 6). This result provides further indication of the significance of *TRP1* prototrophy for tolerance to SDS.

Since SDS treatment activates the CWI pathway [5,6], we wanted to know if tryptophan prototrophy can recover growth inhibition due to other activators of CWI signaling. CFW is a dye that interferes with cell wall assembly by blocking chitin polymerization, resulting in weakened cell walls [5,36,37]. CR is a dye that interferes with cell wall assembly by binding to chitin and cellulose with high affinity [38,39]. Heat stress causes fluidization of the cell membrane and weakens the cell wall [40–42]. TTO, an extract from the leaves of *Melaleuca alternifoli*, is a fungicide that disrupts cell membranes and mitochondrial functions [43,44]. Treatment of yeast with either dye, heat stress or TTO triggers the CWI pathway [9,10,42,45].

We asked whether tryptophan prototrophy could recover growth sensitivity in cells challenged with 10ug/ml CFW or 10ug/ml CR. In contrast to SDS treatment, we found that the different varieties of W303 cells were as sensitive as each other upon CFW or CR treatment and this result was regardless of tryptophan prototrophy (Fig 3B). We also show that BY4741 cells were not as sensitive to CFW and CR as W303 cells (Fig 3B, row 7).

We also tested the effect of tryptophan prototrophy on cells compromised with heat stress. This assay shows that both W303 and BY4741 cells grew well when incubated at 37°C (Fig 3C, left). W303 cell growth was inhibited at 39°C and like CFW and CR treatment; inhibition was completely independent of tryptophan prototrophy (Fig 3C, right). BY4741 cells, however, grew robustly at 39°C (Fig 3C, right, row 7).

In converse to CFW, CR and heat stress, the presence of wild type *TRP1* was able to recover cell growth sensitivity due to TTO in prototrophic cells. We show that W303 prototrophic cells are able to overcome growth inhibition due to 0.15% TTO if they contain *his3-11*,*15*, but not *trp1-1* (Fig 3D, rows 5 and 6). The W303 cells containing several auxotrophic markers could not grow in the presence of 0.15% TTO, nor could the BY4741 cells, indicating that multiple auxotrophies are also detrimental for the response to TTO (Fig 3D, rows 7 and 8). While the effects of TTO are not the same as for SDS, they indicate that tryptophan prototrophy has a similar trend on growth recovery in cells compromised with TTO as with SDS. These results indicate that the activity of the CWI pathway is independent of tryptophan synthesis. Perhaps the stress response involving tryptophan prototrophy is particular to membrane disruptions as opposed to cell wall perturbations.

**Fig 4:**
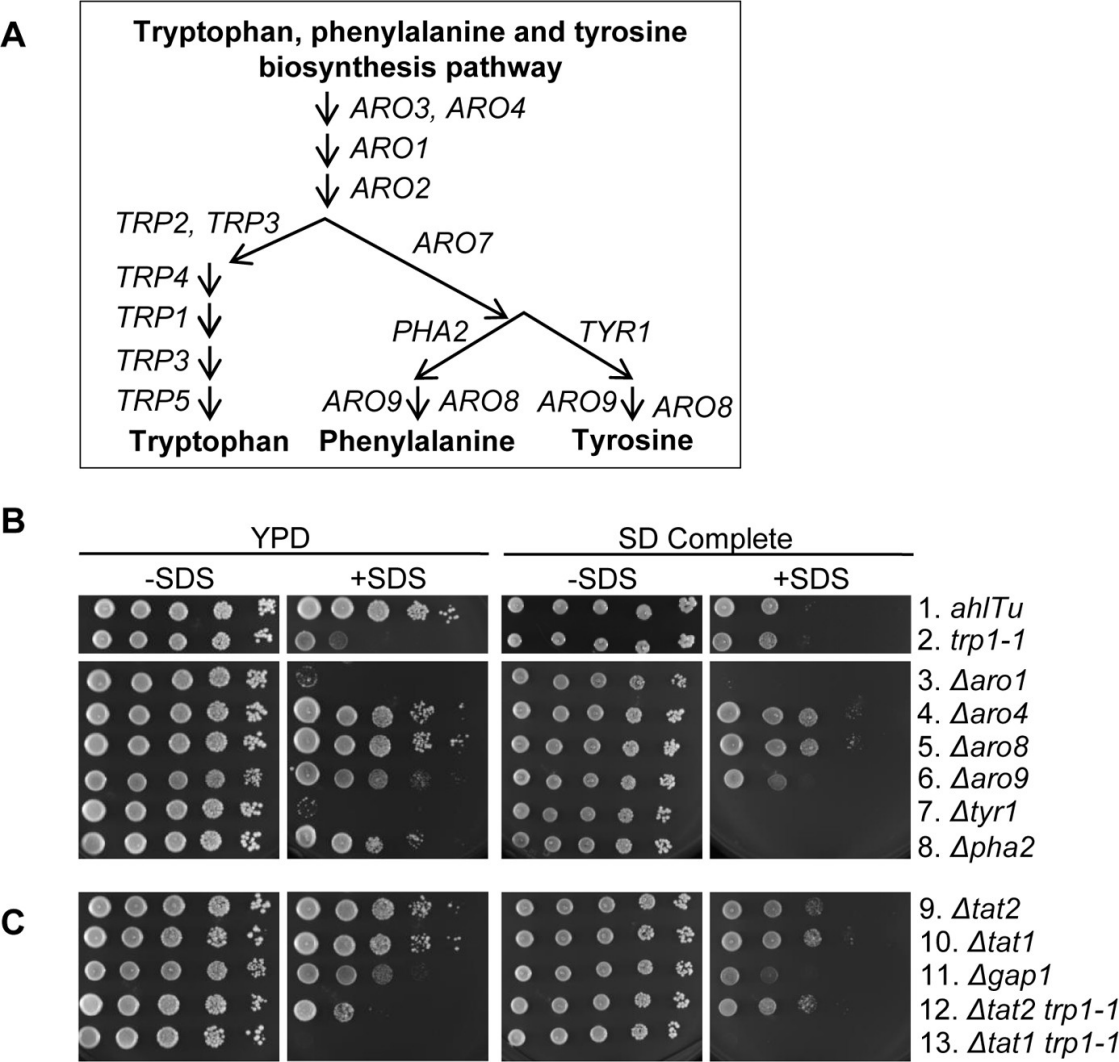
Cells deficient in the biosynthesis of tryptophan or tyrosine are sensitive to SDS. (A) Schematic of the tryptophan, phenylalanine and tyrosine biosynthesis pathway. (B and C) W303 cells with *TRP1* (*ade2-1*, *his3-11*,*15*, *leu2-3*,*112* and *ura3-1)* are labeled as *ahlTu*. W303 cells harboring specified deletions in the tryptophan, phenylalanine and tyrosine biosynthesis pathway were 10-fold serially diluted onto YPD or SD with or without SDS.

The *TRP1* gene product is essential for yeast cells to biosynthesize tryptophan. *S*. *cerevisiae* uses a shared pathway to synthesize tryptophan that also synthesizes phenylalanine and tyrosine (Fig 4A). It is known that BY4741 cells mutant for any of the enzymes specific to the tryptophan branch of this pathway are sensitive to SDS when grown in YPD [35]. We wanted to know how W303 cells defective in the tryptophan, phenylalanine and tyrosine biosynthesis pathway respond to SDS.

To examine this further, we obtained a BY4741 yeast deletion set containing cells harboring a gene deletion for each of the enzymes involved in the tryptophan, phenylalanine and tyrosine biosynthesis pathway (EUROpean *Saccharomyces Cerevisiae* Archive for Functional Analysis (EUROSCARF) [46,47]). Using the BY4741 deletion set, we created several deletion mutants in the W303 background by PCR-based transformation (see Materials and methods).

A serial dilution assay showed that W303 cells defective in tryptophan, phenylalanine or tyrosine biosynthesis grow healthy and robust on YPD while their growth varied with the addition of SDS (Fig 4B, left half). As expected, growth in the W303 *TRP+* cells was not affected by the addition of SDS while growth in the tryptophan auxotroph cells was severely inhibited (Fig 4B, second column, rows 1 and 2). We show that *Δaro1* cell growth was even more inhibited by SDS than the *trp1-1* cells (Fig 4B, second column, row 3), possibly because *ARO1* is involved in both tryptophan and tyrosine biosynthesis. *ARO3* can act in the place of *ARO4* [48]. Indeed, *Δaro4* cells grew robustly on YPD in the presence of SDS (Fig 4B, second column, row 4). *ARO8* and *ARO9* encode enzymes that catalyze the last step in the synthesis of tyrosine and phenylalanine. We show that SDS did not affect growth in the *Δaro8* cells and caused mild growth inhibition in *Δaro9* cells (Fig 4B, second column, rows 5 and 6). In addition, we found that SDS acutely inhibits growth in the cells specific to the tyrosine branch, *Δtyr1* (Fig 4B, second column, row 7). This is in contrast in the phenylalanine specific cells, *Δpha2*, which showed robust growth in the presence of SDS on YPD plates (Fig 4B, second column, row 8). These results demonstrate that tyrosine prototrophy in addition to tryptophan prototrophy is required for yeast cells to survive SDS-treatment when grown on YPD.

We also questioned if the SDS response differs from YPD verses SD. Tryptophan prototrophy did not make cells more resistant to SDS when grown on SD plates confirming the results in Fig 2A (Fig 4B, right). Consistent with the results on YPD, *Δaro1* cells did not grow on SD plates in the presence of SDS (Fig 4B, right half, row 3). *ARO4* and *ARO8* were dispensable for SDS-induced cell growth inhibition due to redundant function of *ARO3* and *ARO9* respectively (Fig 4B, right half, rows 4 and 5). Similar to growth on YPD, *Δtyr1* cell growth was severely inhibited by SDS when grown on SD plates emphasizing the importance of the tyrosine pathway. Interestingly, *Δpha2* cells became sensitive to SDS when grown on SD plates. Fig 3A shows that prototrophic cells grew better in the presence of SDS in general. Taken together, our results indicate that all amino acids may enhance cell growth when encountered with membrane stress at some level but tryptophan and tyrosine play a prominent role.

In the presence of SDS, the BY4741 deletion set recapitulated similar results as the W303 cells when grown on YPD plates but different results when grown on SD plates (S2 Fig). The most striking difference shown was that the BY4741 deletion cells specific to the tryptophan branch were not sensitive to SDS when grown on SD plates. This implicates a difference in the cell background between W303 and BY4741 in the tryptophan biosynthesis pathway. In addition, these results provide additional evidence that nutrients present in rich media affect the SDS response in regard to tryptophan, phenylalanine and tyrosine biosynthesis.

It has been shown before that tryptophan can be imported through channels other than Tat2p, primarily through Gap1p, the General Amino acid Permease [19]. We created *Δtat2* and *Δgap1* deletion cells in the W303 background. In contrast to *trp1-1*, *Δtat2* cell growth was not inhibited by SDS treatment on YPD whereas growth in the *Δgap1* cells was slightly impaired (Fig 4C, left half, rows 9 and 11). We constructed a double mutant, *Δtat2 trp1-1*, which would not be able to import tryptophan through Tat2p nor make its own tryptophan. Growth of the *Δtat2 trp1-1* cells on YPD plates containing SDS was inhibited similarly to the single mutant, *trp1-1* (Fig 4C, left half, row 12). This indicates that tryptophan biosynthesis plays an important role in resistance to SDS and that loss of the *TAT2* receptor does not affect the SDS sensitivity. In this same assay, we tested the growth of *Δtat1* cells. *TAT1* encodes for the Tyrosine high Affinity Transporter. We found that *Δtat1* cell growth was not affected by SDS when grown on YPD suggesting that tyrosine uptake is also remediated by different means during an SDS response (Fig 4C, second column, row 10). However, the double mutant *Δtat1 trp1-1* showed an enhanced growth defect compared to the *trp1-1* single mutant suggesting that both tryptophan and tyrosine contribute to cell growth when cells are challenged with SDS. These results support the idea that internal tryptophan and tyrosine levels are important during an SDS assault on rich medium and that they are acquired through uptake systems other than Tat2p and Tat1p. On synthetic media, our results show that the *Δtat1 trp1-1* double mutant exhibited severe growth defects by SDS treatment (Fig 4C, fourth column, row 13).

In the BY4741 background, *Δtrp1* cell growth was mildly inhibited by SDS when grown on YPD plates (S2 Fig). The *Δtat2*, *Δgap1* and *Δtat2 Δtrp1* cells all grow healthy on YPD plates when challenged with SDS. With the exception of *Δtat1*, all of these cell types, including *Δtrp1* cells, grew healthy on SD plates regardless of the addition of SDS. The BY4741 *Δtat1* cells, however, show mild growth inhibition on YPD and SD plates and show rescue with the addition of SDS. We conclude that tryptophan prototrophy and components in rich media enhance the response to SDS. The difference in the genetic background between W303 and BY4741 might contribute to the phenotypic observation in this experiment. For example, it is known that W303 cells contain a *rad5-G535R* mutation [49].

### SDS treatment enhances tryptophan uptake

**Fig 5:**
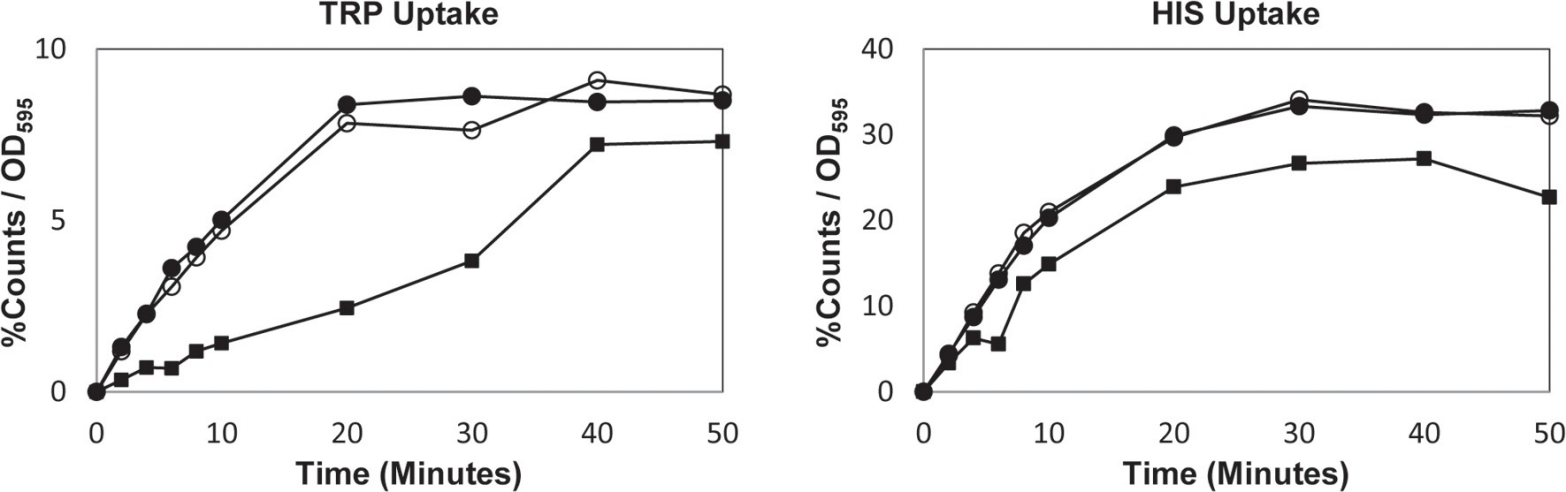
SDS enhances tryptophan uptake. The import rates of radiolabeled tryptophan and histidine were measured using W303 prototrophic cells (*ADE2*, *HIS3*, *LEU2*, *TRP1*, and *URA3)* in the absence (squares) or presence of (open circles) 0.0075% SDS or (solid circles) 0.005% SDS (See Materials and methods). Shown are representative curves.

Since internal tryptophan levels play a more important role than tryptophan uptake in response to SDS, we tested whether membrane disruptions caused by SDS interrupt tryptophan uptake systems. To determine tryptophan uptake, we used prototrophic W303 cells whose growth is uncompromised on YPD plates containing 0.0075% SDS (Fig 3A, row 4). We found that import of radiolabeled L-[5-3H] tryptophan and L-[2,5-3H] histidine was enhanced within minutes upon 0.0075% SDS exposure compared to uncompromised cells in liquid culture (Fig 5). We considered that the enhanced uptake was due to tryptophan and histidine leaking into cells through membrane holes created at this SDS concentration. However, we found the same enhanced uptake when we challenged cells with a lower concentration of SDS (0.005%). These results suggest that cells are not starved for tryptophan or histidine upon SDS administration providing further evidence that the need for tryptophan itself is important. It has been shown that Gap1p activity can be produced within 5 minutes under certain conditions [31,33,34]. It is possible that Gap1p as a high capacity permease is activated by SDS treatment and this is the explanation for increased amino acid uptake.

In summary, we show that the response to SDS triggered cell membrane damage is independent of CWI signaling and is not a cause of tryptophan starvation. Recovering the *TAT2* tryptophan permease from a suppressor gene screen using *Δmck1* cells in the presence of SDS allowed us to examine the biological function of tryptophan during cell membrane stress response due to SDS exposure. First, we show that cells harboring a *trp1-1* mutation have a clear disadvantage in the response to SDS compared to auxotrophies for adenine, histidine, leucine or uracil. Next, we found that tryptophan prototrophy is also critical for stress tolerance towards TTO, another membrane destabilizing drug. While both SDS and TTO cause CWI activation, we demonstrate that tryptophan prototrophy is not able to alleviate growth inhibition due to other cell wall/membrane damaging treatments that also activate the pathway indicating a distinction from CWI signaling. This also implicates that the resistance to growth inhibition shown in tryptophan prototrophic cells may be specific to the type of membrane damage created by SDS and TTO as opposed to cell wall disruptions. In addition, we uncover that tyrosine biosynthesis is also important for resistance to SDS-induced growth inhibition whereas phenylalanine biosynthesis is dispensable. We also found that *Δtat2* deletion cells did not show cell growth sensitivity to SDS implicating that tryptophan levels are maintained during an SDS assault through uptake systems other than Tat2p. Finally, we observe that both tryptophan and histidine import becomes enhanced immediately upon addition of SDS as a further indication that SDS-induced growth inhibition is not due to nutrient starvation in general.

These results suggest that tryptophan, tryptophan biosynthesis and tyrosine biosynthesis play a role in the plasma membrane stress response. It is thought that the constitutive permeases, such as Tat2p and Tat1p, uptake amino acids for use in protein synthesis. Gap1p, however, is a transporter of all amino acids and is regulated by nitrogen [31,32], therefore it is thought that Gap1p acquires amino acids for use as a nitrogen source [50]. While Aro9p is important for the biosynthesis of phenylalanine and tyrosine, it is thought that the main role of Aro9p is to degrade tryptophan [51]. In the W303 cells, we had difficulty making deletions in the tryptophan biosynthesis pathway and were not able to make *Δaro8 Δaro9* double deletion cells. Future directions would be to explore tryptophan and tyrosine biosynthesis and catabolism in respect to nitrogen response pathways and cell membrane damage.

## Materials and methods

### Construction of yeast strains

The yeast cells used are derivatives of W303 or BY4741 (strain list in S1 Table). The LSY112, LSY113, LSY114, LSY115, LSY116, LSY118, LSY119, LSY121, LSY123 and LSY132 cells were made by standard cross and dissection procedures using RUY508 Mat a *his3-11*,*15 leu2-3*,*112 trp1-1 ura3-1 can1-100* (this laboratory) crossed with prototrophic parent cells, Mat α *ade2-1 can1-100*. The haploid genotypes were determined by tetrad analysis. The prototrophic parent cells, kindly provided by Fred Cross, were made in W303 by transformation with the various cloned markers (*ADE2*, *HIS3*, *LEU2*, *TRP1*, and *URA3*) and using mating and tetrad analysis to get haploids with the different combinations. The BY4741 deletion strains were obtained from (EUROSCARF) [46,47]. The deletion set was generated using a PCR based method that deletes each gene from the start to the stop codon with insertion of a KanMX cassette. We confirmed each deletion by PCR using primers A (upstream of start codon) and D (downstream of stop codon) from *Saccharomyces* Genome Deletion Project (Primer list in S2 Table). The W303 deletion mutants were made by standard LiOAc transformation of LSY132 (W303 Mat α *ade2-1 his3-11*,*15 leu2-3*,*112 ura3-1 can1-100*) with PCR products created from the EUROSCARF library deletion strains using *Saccharomyces* Genome Deletion Project primers A and D. The *aro8* W303 cells are an exception and were made with primer D and ARO8 primer A (GAATTGCCATTGATAGAAGAACAGT) designed by our laboratory. The resulting W303 deletion strains were confirmed with primers A and D. The BY4741 or W303 double deletion strains were generated by cross and dissection procedures leading to tetrad analysis followed by PCR confirmation. All phenotypes were confirmed by replica plating to drop out plates (SD-trp, SD-phe, SD-tyr, SD-ade, SD-comp and YPD).

The *TAT2*/pRS425 plasmid was made by PCR of BG1805 YORF (YOLO20W) from the start to the stop codon of *TAT2* using the primers FP1 (CCTGCAGCCCGGGGGATCCA) and RP1 (GGCGGCCGCTCTAGAACTAGTTAACACCAGAAATGGAACT). The *TAT2* PCR product was assembled into the pRS425-2micron *LEU* marked plasmid by Gibson Assembly using the universal primers for pRS425, FP2 (CTAGTTCTAGAGCGGCCGCC) and RP2 (TGGATCCCCCGGGCTGCAGG). The construction of BCY061 (*mck1*::*KanMX)* has been described in [16]. The *TAT2*/pRS425 plasmid or empty vector was transformed into BCY061 using standard lithium acetate transformation and selected on synthetic glucose medium lacking leucine.

### Cell culture and media

Standard procedures were used for yeast extract/peptone/dextrose media (YPD) and synthetic media (SD). The chemicals were added to plates with the final concentrations of 0.0075% SDS, 10ug/ml Calcofluor White (Sigma 18909), 10ug/ml Congo Red, (Sigma C-6767) or 0.15% Tea Tree Oil (Sigma SMB00386). 0.5% Tween 40 was added to the Tea Tree Oil and control plates to assure solubility of the Tea Tree Oil. In Fig 1B and 1D, supplements were spread on top of YPD + SDS plates at 1X final concentration from 100X stocks supplied at 6g/L adenine, 2g/L histidine, 12g/L leucine, 8g/L tryptophan or 2g/L uracil. The plates in Fig 2B contain additional 1X tryptophan (80mg/L), tyrosine (30mg/L) or phenylalanine (50mg/L) supplied to the media. YPD plates were incubated for two days and SD plates for three days at 30°C unless otherwise indicated. Images are representative of three independent experiments.

### Suppressor gene screening

The suppressor gene screen, which has been described in [52], was done using *mck1*::KanMX BCY061 cells transformed with the ATCC YEp13 total yeast genomic DNA library cloned into a 2-micron/*LEU2* high-copy plasmid [53].

### Serial dilution

Cells in Fig 1A were grown overnight at room temperature in synthetic glucose medium lacking leucine and diluted serially 5-fold for five dilutions before plating. 2.5uL spots were used on SDS containing plates otherwise 5uL spots were used. All other serial dilutions were prepared the same method as in Fig 1A except that they were grown in YPD overnight and serially diluted 10-fold.

### Growth and viability assay

In Fig 2B, log phase cells were grown in YPD with or without SDS (0.001%). OD_595_ was read and viability was determined every hour for 4 hours. Viability was assayed with an equivalent of 1mL of cells at OD_595_ = 0.1, washed with PBS pH 7.4, sonicated, stained with propidium iodide and read using BD Accuri C6 flow cytometer (BD Biosciences, NJ) with the parameters FL2 and FSC-H. Live and dead cells were determined via gating. Doubling times as the slope of the line was determined by plotting log2 of the OD reading vs time.

### Amino acid uptake assay

The protocol was adapted from J Heitman [54]. LSY119 cells in log-phase were harvested and washed once with 10mM sodium citrate, pH4.5, and resuspended in 50mLs of 10mM sodium citrate, pH4.5, containing 20mM ammonium sulfate and 2% glucose. SDS at 0.0075% or 0.005% was added and the 0 time point was taken immediately before the radioactive substrate addition. Uptake was assayed by adding 0.5mL of radiolabeled amino acid mixture to 4.5mL cell culture. The radiolabeled amino acid mixture was made with 495.5uL H_2_O and 4.5uL of either tryptophan, L-[5-3H], 20 Ci/mmol, or histidine, L-[ring-2,5-3H], 50 Ci/mmol (American Radiolabeled Chemicals, Inc.). Aliquots of 0.5mL were taken at 0 time point (no radioactivity) and in 2 min intervals from 2–10 min and then every 10 min up to 50 min. Cells were vacuum filtered onto Whatman glass microfiber filters (Sigma WHA1825025) presoaked in 10mM sodium citrate, pH4.5, and washed twice with 2mL 10mM sodium citrate, pH4.5, containing 2mM tryptophan and histidine. Filters were dried and the remaining radioactivity was quantified with 5mL scintillation fluid. Percent counts were normalized by cell density determined by OD_595_.

### Reagent availability

All strains and protocols are available upon request.

## Supporting information

S1 Fig *TAT2* does not rescue SDS sensitivity in *Δmck1* cells when grown on synthetic media. W303 *MCK1* cells (*his3-11*,*15 leu2-3*,*112 trp1-1 ura3-1*) and isogenic *Δmck1* cells transformed with pRS425 empty plasmid or *TAT2*/pRS425 plasmid were 10-fold serially diluted onto SD-leu with or without 0.0075% SDS.
(PDF)

S2 Fig BY4741 cells deficient in the biosynthesis of tryptophan and tyrosine are sensitive to SDS. BY4741 wild-type cells (WT) (*his3Δ1 leu2Δ0 met15Δ0 ura3Δ0)* and cells harboring the indicated deletions in the tryptophan, phenylalanine and tyrosine biosynthesis pathway were 10-fold serially diluted onto YPD or SD with or without 0.0075% SDS.
(PDF)

S1 Table *S*. *cerevisiae* cell types used in this study. (PDF)

S2 Table *Saccharomyces* Genome Deletion Project primer sequences. (PDF)
