# Supplementary figures and images for: Tryptophan confers resistance to SDS-associated cell membrane stress in Saccharomyces cerevisiae
Source: PLoS One. 2019 Mar 11;14(3):e0199484. doi: 10.1371/journal.pone.0199484 (PMC6411118; doi:10.1371/journal.pone.0199484)

**S1 Fig**

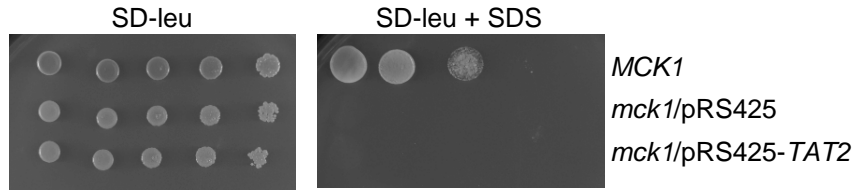

Supplement: S1 Fig — W303 MCK1 cells (his3-11,15 leu2-3,112 trp1-1 ura3-1) and isogenic Δmck1 cells transformed with pRS425 empty plasmid or TAT2/pRS425 plasmid were 10-fold serially diluted onto SD-leu with or without 0.0075% SDS. (PDF) [file pone.0199484.s001.pdf]

S2 Fig

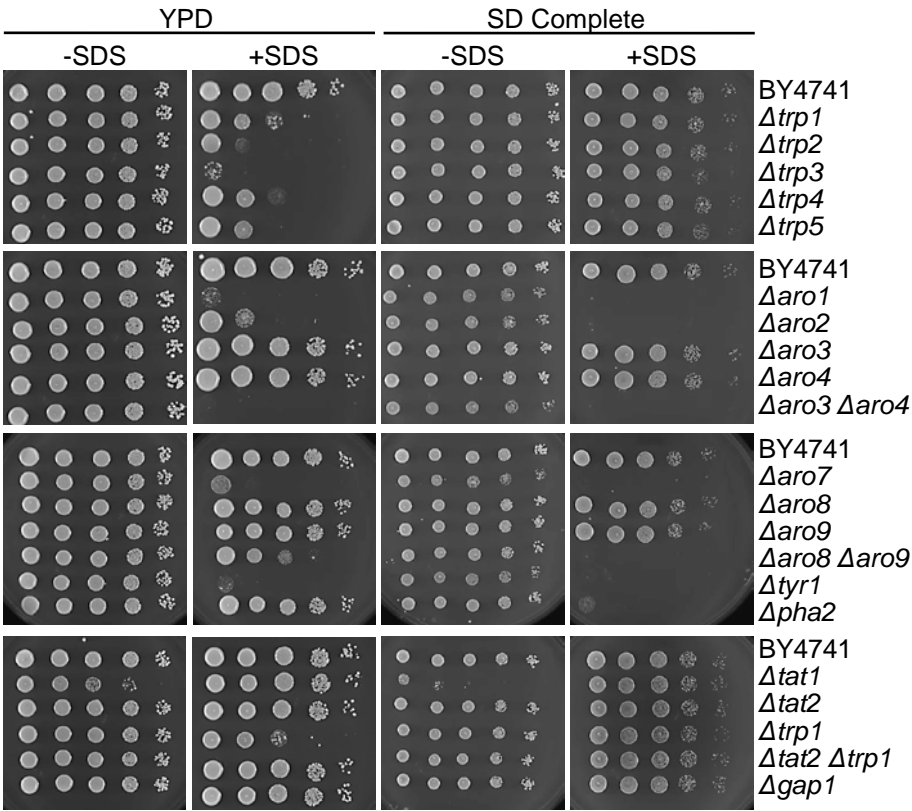

Supplement: S2 Fig — BY4741 wild-type cells (WT) (his3Δ1 leu2Δ0 met15Δ0 ura3Δ0) and cells harboring the indicated deletions in the tryptophan, phenylalanine and tyrosine biosynthesis pathway were 10-fold serially diluted onto YPD or SD with or without 0.0075% SDS. (PDF) [file pone.0199484.s002.pdf]
