# Supplementary material for: Tryptophan confers resistance to SDS-associated cell membrane stress in Saccharomyces cerevisiae
Source: PLoS One. 2019 Mar 11;14(3):e0199484. doi: 10.1371/journal.pone.0199484 (PMC6411118; doi:10.1371/journal.pone.0199484)
Supplement: S1 Table — (PDF) [file pone.0199484.s003.pdf]

S1 Table. *S. cerevisiae* cell types used in this study.

Strain	Genotype	Source
RUY508	MATa *his3-11,15 leu2-3,112 trp1-1 ura3-1 can1-100*	This Laboratory
BCY061	MATa *mck1::KanMX his3-11,15 leu2-3,112 trp1-1 ura3-1 can1-100*	[13]
LSY183	MATa *trp1::KanMX his3Δ1 leu2Δ0 met15Δ0 ura3Δ0*	Euroscarf
RUY120	MATa *ade2-1 his3-11,15 leu2-3,112 trp1-1 ura3-1 can1-100*	This Laboratory
RUY064	MATa *his3Δ1 leu2Δ0 met15Δ0 ura3Δ0*	This Laboratory
RUY065	MATα *his3Δ1 leu2Δ0 met15Δ0 ura3Δ0*	This Laboratory
LSY119	MATa *can1-100*	This Laboratory
LSY123	MATa *trp1-1 can1-100*	This Laboratory
LSY116	MATa *ade2-1 his3-11,15 leu2-3,112 ura3-1 can1-100*	This Laboratory
LSY112	MATa *ade2-1 his3-11,15 leu2-3,112 trp1-1 ura3-1 can1-100*	This Laboratory
LSY113	MATa *his3-11,15 leu2-3,112 trp1-1 ura3-1 can1-100*	This Laboratory
LSY114	MATa *ade2-1 leu2-3,112, trp1-1 ura3-1 can1-100*	This Laboratory
LSY115	MATa *ade2-1 his3-11,15 trp1-1 ura3-1 can1-100*	This Laboratory
LSY118	MATa *ade2-1 his3-11,15 leu2-3,112 trp1-1 can1-100*	This Laboratory
LSY121	MATa *his3-11,15 can1-100*	This Laboratory
LSY176	MATa *aro1::KanMX his3Δ1 leu2Δ0 met15Δ0 ura3Δ0*	Euroscarf
LSY177	MATa *aro2::KanMX his3Δ1 leu2Δ0 met15Δ0 ura3Δ0*	Euroscarf
LSY178	MATa *aro3::KanMX his3Δ1 leu2Δ0 met15Δ0 ura3Δ0*	Euroscarf
LSY179	MATa *aro4::KanMX his3Δ1 leu2Δ0 met15Δ0 ura3Δ0*	Euroscarf

LSY203	MATa *aro3::KanMX aro4::KanMX his3Δ1 leu2Δ0 met15Δ0 ura3Δ0*	This Laboratory
LSY204	MATa *aro3::KanMX aro4::KanMX his3Δ1 leu2Δ0 met15Δ0 ura3Δ0*	This Laboratory
LSY180	MATa *aro7::KanMX his3Δ1 leu2Δ0 met15Δ0 ura3Δ0*	Euroscarf
LSY181	MATa *aro8::KanMX his3Δ1 leu2Δ0 met15Δ0 ura3Δ0*	Euroscarf
LSY182	MATa *aro9::KanMX his3Δ1 leu2Δ0 met15Δ0 ura3Δ0*	Euroscarf
LSY208	MATa *aro8::KanMX aro9::KanMX his3Δ1 leu2Δ0 met15Δ0 ura3Δ0*	This Laboratory
LSY209	MATa *aro8::KanMX aro9::KanMX his3Δ1 leu2Δ0 met15Δ0 ura3Δ0*	This Laboratory
LSY183	MATa *trp1::KanMX his3Δ1 leu2Δ0 met15Δ0 ura3Δ0*	Euroscarf
LSY184	MATa *trp2::KanMX his3Δ1 leu2Δ0 met15Δ0 ura3Δ0*	Euroscarf
LSY185	MATa *trp3::KanMX his3Δ1 leu2Δ0 met15Δ0 ura3Δ0*	Euroscarf
LSY186	MATa *trp4::KanMX his3Δ1 leu2Δ0 met15Δ0 ura3Δ0*	Euroscarf
LSY187	MATa *trp5::KanMX his3Δ1 leu2Δ0 met15Δ0 ura3Δ0*	Euroscarf
LSY188	MATa *tyr1::KanMX his3Δ1 leu2Δ0 met15Δ0 ura3Δ0*	Euroscarf
LSY189	MATa *pha2::KanMX his3Δ1 leu2Δ0 met15Δ0 ura3Δ0*	Euroscarf
LSY200	MATa *tat2::KanMX trp1::KanMX his3Δ1 leu2Δ0 met15Δ0 ura3Δ0*	This Laboratory
LSY210	MATa *gap1::KanMX his3Δ1 leu2Δ0 met15Δ0 ura3Δ0*	Euroscarf
LSY132	MATα *ade2-1 his3-11,15 leu2-3,112 ura3-1 can1-100*	This Laboratory
LSY220	MATα *aro1::KanMX ade2-1 his3-11,15 leu2-3,112 ura3-1 can1-100*	This Laboratory

LSY224	MATα *aro4::KanMX ade2-1 his3-11,15 leu2-3,112 ura3-1 can1-100*	This Laboratory
LSY240	MATα *aro8::KanMX ade2-1 his3-11,15 leu2-3,112 ura3-1 can1-100*	This Laboratory
LSY226	MATα *aro9::KanMX ade2-1 his3-11,15 leu2-3,112 ura3-1 can1-100*	This Laboratory
LSY232	MATα *tyr1::KanMX ade2-1 his3-11,15 leu2-3,112 ura3-1 can1-100*	This Laboratory
LSY233	MATα *pha1::KanMX ade2-1 his3-11,15 leu2-3,112 ura3-1 can1-100*	This Laboratory
LSY234	MATα *tat1::KanMX ade2-1 his3-11,15 leu2-3,112 ura3-1 can1-100*	This Laboratory
LSY235	MATα *tat2::KanMX ade2-1 his3-11,15 leu2-3,112 ura3-1 can1-100*	This Laboratory
LSY241	MATα *gap1::KanMX his3-11,15 leu2-3,112 ura3-1 can1-100*	This Laboratory
LSY215	MATa *tat2::KanMX ade2-1 his3Δ1 leu2Δ0 trp1-1 ura3Δ0 can1-100*	This Laboratory
LSY214	MATa *tat1::KanMX ade2-1 his3Δ1 leu2Δ0 trp1-1 ura3Δ0 can1-100*	This Laboratory
