# Supplementary material for: Tryptophan confers resistance to SDS-associated cell membrane stress in Saccharomyces cerevisiae
Source: PLoS One. 2019 Mar 11;14(3):e0199484. doi: 10.1371/journal.pone.0199484 (PMC6411118; doi:10.1371/journal.pone.0199484)
Supplement: S2 Table — (PDF) [file pone.0199484.s004.pdf]

S2 Table. ***Saccharomyces*** **Genome Deletion Project primer sequences.**

Gene name	ORF name	Primer sequences
TRP1	YDR007W	**A**: AGAGACCAATCAGTAAAAATCAACG<br>**D**: GCGAAAAGACGATAAATACAAGAAA
TRP2	YER090W	**A**: CCTTTCAATCGTTGAAGTAGTTTGT<br>**D**: ACATCGGTGATTTAATTTTTGTGTT
TRP3	YKL211C	**A**: AGGCCTTTTTGAACTATTTTCTGTT<br>**D**: AAAGGTTTGAAATATGCACAGAGTC
TRP4	YDR354W	**A**: ATGACTAATATTATTGCTGCGCTTC<br>**D**: TTATTGGCATTTGGAACCTTAGTAG
TRP5	YGL026C	**A**: CAGTACAATATGAATATGGGCATGA<br>**D**: ATTCGTGAACTTTTGATATTCTTCG
ARO1	YDR127W	**A**: CTACGACCATAAGCACTACAACCTT<br>**D**: ACTTTCAACGATAATGATTTTCCAA
ARO2	YGL148W	**A**: CCAAAAAGAAGTGTCTTTGATGACT<br>**D**: ACGAAATTGTCTTGTCAGGTAACTC
ARO3	YDR035W	**A**: TTTAAAAGCTTCCTCACTTTCCTTT<br>**D**: GGTATCCAATCGCTATGTAAAATTG
ARO4	YBR249C	**A**: CATTGTTAGCTCATTGAATACATCG<br>**D**: TTCAATAGCTGCCTCTTTTCTTTTA
ARO7	YPR060C	**A**: AGCAGCTAAATGAAATCACCTATTG<br>**D**: CAAACGGATAATTTACAAAAGCCTA
ARO8	YGL202W	**A**: GAATTGCCATTGATAGAAGAACAGT<br>**D**: GATCTTGAGAATGAGGAAAATGAAA
ARO9	YHR137W	**A**: CAATCAGAGAGAAGTTGAAGAGGAG<br>**D**: GGCTTTACAGTGGACTTACCTGATA
TYR1	YBR166C	**A**: TGATGTTGAAGATGACGATAAAGAA<br>**D**: GACACATGACTACAATGCTATCGAC
PHA2	YNL316C	**A**: AGAAACTCCAGTTGCTAAACAGAGA<br>**D**: AATGCTGTGAGAGAGAAGAGTGAAT
TAT1	YBR069C	**A**: AAACTTCACATTATCTTGACAAGGC<br>**D**: TTTTCTTGGCACATTTACACACTTA
TAT2	YOL020W	**A**: GGTGTATCGTTAAATGGTACGTAGG<br>**D**: TAAATTACAGTCTTGCGCCTTAAAC
GAP1	YKR039W	**A**: GTTAAATGTCAGTTTGGATGCTTTT<br>**D**: GGGAAATCATATTGATTGATTGAAG
